# Will epigenetics be a key player in crop breeding?

**DOI:** 10.3389/fpls.2022.958350

**Published:** 2022-09-30

**Authors:** Kaoru Tonosaki, Ryo Fujimoto, Elizabeth S. Dennis, Victor Raboy, Kenji Osabe

**Affiliations:** ^1^ Kihara Institute for Biological Research, Yokohama City University, Yokohama, Japan; ^2^ Graduate School of Agricultural Science, Kobe University, Kobe, Japan; ^3^ Commonwealth Scientific and Industrial Research Organisation (CSIRO) Agriculture and Food, Canberra, ACT, Australia; ^4^ School of Life Sciences, Faculty of Science, University of Technology Sydney, Ultimo, NSW, Australia; ^5^ Independent Researcher Portland, Portland, OR, United States; ^6^ Institute of Scientific and Industrial Research (SANKEN), Osaka University, Osaka, Japan

**Keywords:** DNA methylation, breeding, intergenerational inheritance, transgenerational inheritance, epigenetics, epiallele, epigenome editing, paramutation

## Abstract

If food and feed production are to keep up with world demand in the face of climate change, continued progress in understanding and utilizing both genetic and epigenetic sources of crop variation is necessary. Progress in plant breeding has traditionally been thought to be due to selection for spontaneous DNA sequence mutations that impart desirable phenotypes. These spontaneous mutations can expand phenotypic diversity, from which breeders can select agronomically useful traits. However, it has become clear that phenotypic diversity can be generated even when the genome sequence is unaltered. Epigenetic gene regulation is a mechanism by which genome expression is regulated without altering the DNA sequence. With the development of high throughput DNA sequencers, it has become possible to analyze the epigenetic state of the whole genome, which is termed the epigenome. These techniques enable us to identify spontaneous epigenetic mutations (epimutations) with high throughput and identify the epimutations that lead to increased phenotypic diversity. These epimutations can create new phenotypes and the causative epimutations can be inherited over generations. There is evidence of selected agronomic traits being conditioned by heritable epimutations, and breeders may have historically selected for epiallele-conditioned agronomic traits. These results imply that not only DNA sequence diversity, but the diversity of epigenetic states can contribute to increased phenotypic diversity. However, since the modes of induction and transmission of epialleles and their stability differ from that of genetic alleles, the importance of inheritance as classically defined also differs. For example, there may be a difference between the types of epigenetic inheritance important to crop breeding and crop production. The former may depend more on longer-term inheritance whereas the latter may simply take advantage of shorter-term phenomena. With the advances in our understanding of epigenetics, epigenetics may bring new perspectives for crop improvement, such as the use of epigenetic variation or epigenome editing in breeding. In this review, we will introduce the role of epigenetic variation in plant breeding, largely focusing on DNA methylation, and conclude by asking to what extent new knowledge of epigenetics in crop breeding has led to documented cases of its successful use.

## Introduction

**Figure 1:**
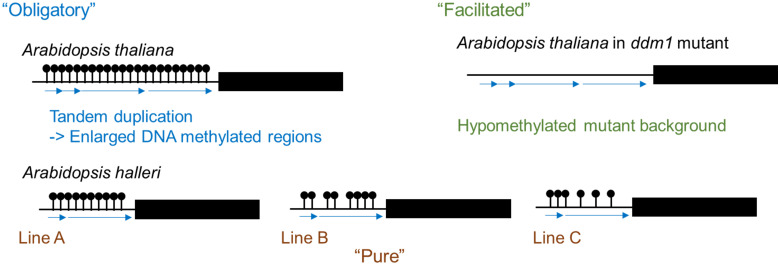
Three classes of epigenetic variation. Variation of DNA methylation results in the formation of tandem repeats (“Obligatory”). Demethylation is observed in hypomethylated mutants such as ddm1 (“Facilitated”). Natural variations in DNA methylation levels even without any DNA sequence differences between lines (“Pure”).

DNA methylation refers to an addition of a methyl group at the fifth carbon position of a cytosine ring and is one epigenetic mechanism. In plants, DNA methylation is observed in the symmetric CG and CHG contexts as well as the asymmetric CHH context (where H is A, C, or T). DNA methylation states are stably inherited and play a role in transcriptional regulation not only of protein coding genes but also of transposable elements (TEs) (Fujimoto et al., 2012; Kawakatsu and Ecker, 2019). Intraspecific variation of DNA methylation states indicates the occurrence of spontaneous changes in DNA methylation. Three classes of epigenetic variation have been defined (Richards, 2006). “Obligatory” epigenetic variation is completely dependent on DNA sequence change; an example of this is transposon-associated epigenetic change. “Facilitated” epigenetic variation is caused by stochastic variation in epigenetic states and change of DNA methylation state in hypomethylated mutants is an example. “Pure” epigenetic variation is generated stochastically and is completely independent of DNA sequence (Richards, 2006). In the genus Arabidopsis, there is a variation in the number of tandem repeats in the promoter region of *FLOWERING WAGENINGEN* (*FWA*) between related species, and DNA methylated regions are enlarged by the generation of tandem repeats. Therefore, this falls under “obligatory” epigenetic variation (**Figure 1**) (Fujimoto et al., 2008). In *Arabidopsis thaliana*, the promoter region of *FWA* is highly methylated and *FWA* expression is silenced, while in the hypomethylated mutants, there is no DNA methylation in the promoter region and *FWA* is expressed causing delayed flowering (Soppe et al., 2000). The late flowering phenotype and hypomethylated state in the promoter region were stably inherited to the next generation (Kakutani, 1997), and this epigenetic variation falls under “facilitated” epigenetic variation (**Figure 1**). There is a natural variation of DNA methylation states without DNA sequence polymorphism between accessions of the same species and this epigenetic variation falls under the “pure” epigenetic variation (**Figure 1**) (Fujimoto et al., 2008; Fujimoto et al., 2011).

Studies using mutants of genes with DNA methylation or demethylation functions have revealed various biological roles of DNA methylation (Fujimoto et al., 2012; Kumar and Mohapatra, 2021). In hypomethylated mutants of *A. thaliana*, developmental abnormalities and transposition of transposons were observed. Some developmental abnormalities in *A. thaliana* were due to the loss of function by insertion of endogenous transposons in genes or their regulatory regions (Miura et al., 2001; Mirouze et al., 2009; Tsukahara et al., 2009), and some were due to a change of gene expression caused by changes in epigenetic states resulting from transposable insertion in a nearby gene (Saze and Kakutani, 2007; Fujimoto et al., 2008). Loss-of-function mutants of genes orthologous to those in *A. thaliana* involved in DNA methylation have been analyzed in crops. The effects are generally similar to those in *A. thaliana*, but phenotypic changes or defects can be seen for some mutants of crops that do not show any phenotypic changes in the orthologous mutant of *A. thaliana* (Moritoh et al., 2012; Hu et al., 2014; Li et al., 2014; Yamauchi et al., 2014; Cheng et al., 2015; Tan et al., 2016; Corem et al., 2018; Grover et al., 2018; Xu et al., 2020a; Zhang et al., 2020).

It has become possible to examine DNA methylation states at single base pair resolution; differences in DNA methylation between accessions, tissue specificity of DNA methylation, frequency of change of DNA methylation, and heritability of altered DNA methylation have been observed (Kawakatsu and Ecker, 2019; Kumar and Mohapatra, 2021). These studies have shown that a change of DNA methylation can cause changes in plant traits, and some traits caused by change of DNA methylation are heritable over generations (Kawakatsu and Ecker, 2019; Kumar and Mohapatra, 2021). Whole-genome bisulfite sequencing (WGBS) is commonly used for the identification of DNA methylation states, and this method needs a reference genome. Sequencing innovations have led to determining the whole-genome sequence in many crops, increasing the number of crops that can be analyzed by WGBS. Even when whole-genome sequence information is not available, alternative methods such as methylation-sensitive amplified polymorphism (MSAP) or bisulfite-converted restriction site-associated DNA sequencing (BsRADseq) enable us to examine the DNA methylation state (Trucchi et al., 2016).

A heritable change of epigenetic state that produces new epigenetic alleles (epialleles) is an epimutation, and a heritable phenotypic change caused by epimutations/epialleles is called an epimutant. Inheritance of epigenetic alterations in natural populations or breeding populations needs to be evaluated to understand how epimutants contribute to evolution or crop domestication (Richards, 2008). The importance of epimutations/epialleles in crop breeding depends on whether the epimutations/epialleles are the cause of the phenotypic variation and whether these epimutations/epialleles and their associated traits are stably inherited over generations. Increasing knowledge about heritable epigenetic changes associated with phenotypic variations foreshadows the importance of epimutations/epialleles in crop breeding. While care must be taken to properly identify and document what is or isn’t a truly heritable epimutation that is independent of cryptic DNA sequence changes, or of maternal effects, there is evidence of selected agronomic traits being caused by heritable epimutations in the absence of DNA sequence change or maternal effects. With that said, it is possible that breeders have historically selected for agronomically useful traits caused by epimutations. In this review, we describe the possibility of epimutations/epialleles contributing to phenotypic variation and discuss the potential applications of epigenetic changes in crop breeding. Since both the modes of induction and transmission of epialleles, and their stability differ greatly from that of genetic alleles, the importance of “inheritance” as classically viewed also differs. For example, there may be a difference in the types or duration of epigenetic inheritance important to crop breeding versus crop production. The former may depend more heavily on longer-term forms of epigenetic inheritance whereas the latter may simply take advantage of frequently observed shorter-term epigenetic phenomena.

## Epigenetics and crop breeding

### Basic mechanism of epigenetic gene regulation

A great deal of progress has been made in studying the molecular mechanisms underlying the induction, maintenance, and transmission of epigenetic variation in the model plant *A. thaliana*. Its relatively small genome size, relatively simple genome structure, and ease of use for research purposes makes it an excellent model species and the knowledge gained in studies of *A. thaliana* is largely translatable to crops. While the genomes of most crops are much larger and more complex than that of *A. thaliana*, the genic complements of all higher plant species are relatively similar and the molecular biology of epigenetics is largely conserved across *A. thaliana* and cultivated crops. However, there are important differences (Springer et al., 2016). These largely relate to the role of epigenetics in dealing with the effects of TEs in the genome duplication events that have led to the large genome sizes of crops. So, while any review of epigenetics will be of necessity to include discussion of results from studies of *A. thaliana*, we will also endeavor to review those aspects of epigenetics studied in detail in crops, or studies of aspects of epigenetics that are clearly important to crops. These include studies of paramutation in maize (*Zea mays* L.) and other crop species, and studies of inter- and transgenerational inheritance that is often important for short-term response or adaptation to environmental changes and stress in crop plants.

DNA methylation is the best-known epigenetic modification important for transcriptional regulation. There are two types of this modification: maintenance of DNA methylation and *de novo* DNA methylation. In maintenance of DNA methylation, during replication, methylated DNA strands are separated, and a new unmethylated daughter strand is synthesized. Hemi-methylated DNA is recognized by maintenance DNA methyltransferases that methylate the unmethylated cytosine (Kawakatsu and Ecker, 2019). *De novo* DNA methylation is mediated by RNA-directed DNA methylation (RdDM) coordinating with 24 nucleotide small interfering RNA (24nt-siRNA). DNA sequences complementary to the 24nt-siRNA that have been generated are methylated (Matzke and Mosher, 2014). Many players involved in DNA methylation have been identified, especially by identifying the causative genes for the hypomethylated mutants of *A. thaliana*. Loss-of-function mutants of METHYLTRANSFERASE 1 (MET1), CHROMOMETHYLASE 3 (CMT3), and CMT2 have been shown to be predominantly responsible for maintaining DNA methylation of CG, CHG, and CHH contexts, respectively, and DOMAINS REARRANGED METHYLASE 2 (DRM2) in RdDM (Kawakatsu and Ecker, 2019). In addition to DNA methyltransferases, a methyl-cytosine binding protein such as VARIANT IN METHYLATION 1 (VIM1), histone methyltransferases such as KRYPTONITE (KYP)/SUPPRESSOR OF VARIEGATION 3-9 HOMOLOG 4 (SUVH4), histone deacetylases such as HISTONE DEACETYLASE 6 (HDA6), and chromatin remodelers such as Decrease in DNA methylation 1 (DDM1) play a role in DNA methylation. Active DNA demethylation is mediated by the protein family having bifunctional glycosylase/lyase activity such as DEMETER (DME), DEMETER-LIKE 1/REPRESSOR OF SILENCING 1 (DML1/ROS1), DML2, and DML3 in *A. thaliana* (Zhang et al., 2018a; Kawakatsu and Ecker, 2019).

Histone modification is another epigenetic modification. Histone octamers consist of two of each of the core histones H2A, H2B, H3, and H4, around which wraps ~146bp of DNA, forming a nucleosome that is a basic unit of chromatin. Chromatin structure dictates transcriptional activity and inactivity, and post translational histone modifications including methylation, acetylation, phosphorylation, and ubiquitination of the N-terminal tails of the core histones are involved in alteration of chromatin structure. Generally, adding an acetyl group to a lysine residue of histones by histone acetyltransferase (HAT) is associated with transcriptional activation, while removal of the acetyl group by histone deacetylase (HDAC) is associated with transcriptional repression. In the case of histone methylation, lysine residues are methylated and the number of methyl groups (e.g., mono-, di-, or tri-methylation) is typically associated with transcriptional activation or repression. These changes are catalyzed by the histone lysine methyltransferase (HKMTase) and histone demethylases (HDMases). For example, trimethylation of lysine 4 of histone H3 (H3K4me3) and H3K36me3 are often associated with transcriptional activation, while H3K9me2 and H3K27me3 are associated with transcriptional repression (He et al., 2011; Quadrana and Colot, 2016; Talbert and Henikoff, 2021).

### Comparison of classical vs. epigenetic breeding

The basic concept of breeding is the selection of superior individuals from a population. Selected superior traits should be inherited in a stable manner from one generation to the next. Genetic study has identified many causative genes for agriculturally important traits that have been selected, and many of them are due to differences of nucleotide sequences such as nonsense mutations (Meyer and Purugganan, 2013). Heritable phenotypic variation is caused by genetic diversity, but it is also caused by epigenetic factors including DNA methylation. For example, a change of flower structure from fundamental symmetry to radial symmetry (peloric) in *Linaria vulgaris* is due to transcriptional repression of the *Lcyc* gene by DNA methylation in its genic region (Cubas et al., 1999). Another example is the *colorless non-ripening* (*cnr*) mutant in tomato (*Solanum lycopersicum* L.). Non-ripening of tomato fruit is due to the silencing of the *LeSPL-CNR* gene caused by high levels of DNA methylation in its promoter region (Manning et al., 2006). In these two cases, there is no sequence polymorphism in causative genes between the wild type and mutants, indicating that spontaneous epimutation causes heritable phenotypic change (Cubas et al., 1999; Manning et al., 2006).

In addition to the two examples of single-gene, qualitative traits mentioned above, the involvement of an epigenetic component has also been documented for quantitative traits. Selection of energy use efficiency (EUE), which is an important factor in determining canola (*Brassica napus* L.) yield, is solely based on epigenetic components, as a genetically identical canola population was used for selection. The epigenetic EUE component was stably inherited, which allowed for selection (Hauben et al., 2009). This initial work with canola was followed up with a similar approach in rice (*Oryza sativa* L.); recurrent selection for EUE resulted in enhanced yield in field trials which again was due to heritable epigenetic change (Schmidt et al., 2018).

A second example of an approach to breeding for enhanced yield that takes advantage of epigenetics involves silencing of *MutS HOMOLOG1* (*MSH1*) *via* RNA interference (RNAi) (Yang et al., 2015; Yang et al., 2020). Silencing of *MSH1* induces heritable epigenomic changes, termed “methylation repatterning”, a process that requires siRNA and the RdDM pathway. This repatterning increases phenotypic plasticity. Following removal of the RNAi suppression *via* outcrossing and selection of transgene-null segregants, lines with stably enhanced growth and vigor can be identified. This has been documented in tomato (Yang et al., 2015), soybean [*Glycine max* L. (Merr)] (Raju et al., 2018), and sorghum (*Sorghum bicolor* L. Moench) (Ketumile et al., 2022). These results indicate that some epimutations/epialleles and epigenomic states that condition agriculturally-important traits are heritable and it is conceivable that they have played a wider role in crop breeding than previously known.

The development of the WGBS method that enables the detection of DNA methylation at single base pair resolution has increased the accuracy of epimutation detection (Kawakatsu and Ecker, 2019). Comparison of DNA methylation states between more than 1,000 *A. thaliana* accessions determined 78% of methylated cytosines were differentially methylated across accessions. DNA methylation is correlated with place of origin and its climate, suggesting it plays a role in plant adaptation (Kawakatsu et al., 2016a). Differences in DNA methylation states between accessions have been identified in maize, rice, soybean, etc. (He et al., 2010; Shen et al., 2018; Xu et al., 2020b). A comparison of DNA methylation states between parental lines and their progenies generated from single seed descent over 30 generations showed that the rate of spontaneous changes of DNA methylation (4x10^-4^ methylation polymorphisms per CG site per generation) is four to five orders of magnitude higher than the rate of spontaneous genetic mutations (7x10^-9^ base substitutions per site per generation) (Ossowski et al., 2010; Schmitz et al., 2011).

The extent to which epimutation can play an important role in crop breeding depends on the heritability of epimutations and their ability to produce new phenotypic change. Using two recombinant inbred lines from two different *A. thaliana* accessions, five generations of selection produced variations in flowering time and plant architecture within a population, and changed phenotypes were stably inherited for two to three generations. These variations resulted in altered DNA methylation because of the presence of a small number of single nucleotide polymorphisms (SNPs) between selected individuals and their original plants (Schmid et al., 2018). In maize, using 263 inbred genotypes, the association between phenotypic diversity of metabolic traits and differentially methylated regions not including any SNPs, suggested that DNA methylation can cause phenotypic variation (Xu et al., 2019). Unlike DNA sequence, *trans* acting DNA methylation change was identified in F_1_ hybrids derived from crossing between two different accessions of *A. thaliana*, though *cis* acting DNA methylation was the majority (Greaves et al., 2012a; Zhang et al., 2016). Two mechanisms causing nonadditive DNA methylation states in F_1_ hybrids were proposed; *trans*-chromosomal methylation (TCM) that is due to an increase in DNA methylation at a locus with a previously low methylation allele gaining methylation to resemble the more heavily methylated allele, and *trans*-chromosomal demethylation (TCdM) that is due to loss of DNA methylation at one of the methylated genomic segments (Greaves et al., 2012a; Greaves et al., 2012b; Fujimoto et al., 2018). siRNAs have the ability to modify epigenetic marks in *trans*; siRNA derived from one parental allele can modify the DNA methylation state in the other parental allele (Groszmann et al., 2013; Greaves et al., 2015). Indeed, RdDM is involved in nonadditive DNA methylation states in F_1_ hybrids (Zhang et al., 2016). Some nonadditive DNA methylation states were heritable in the F_2_ generation, though there were variations of DNA methylation levels among individual F_2_ plants (Greaves et al., 2016). These results suggest that in crossbreeding populations there will be greater variation in DNA methylation states than DNA sequences, and this may generate phenotypic diversity.

**Figure 2:**
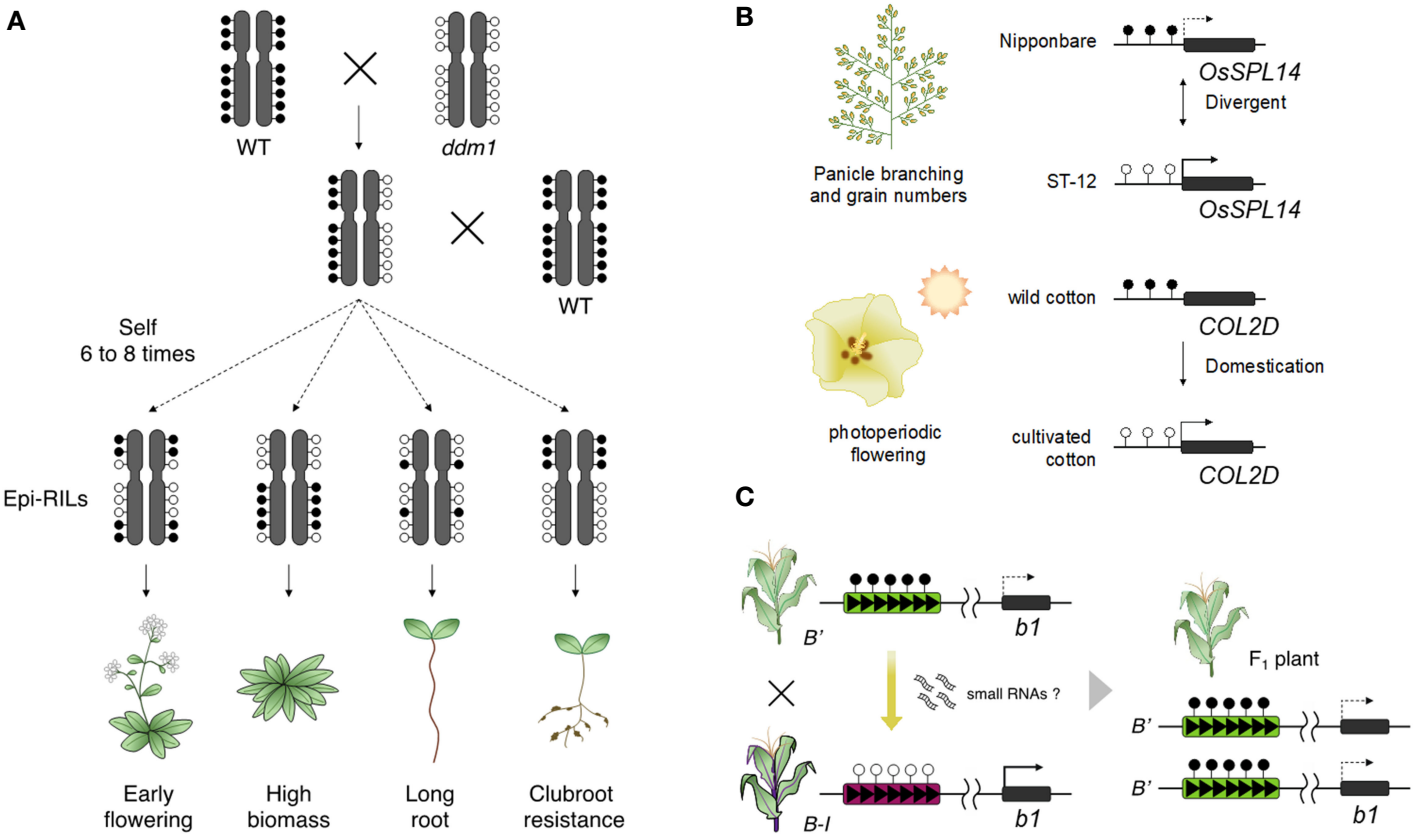
Epigenetic changes lead to phenotypic diversity. **(A)** Epi-RILs shows phenotypic changes in agronomic traits such as early flowering, increased plant growth, root length, and clubroot resistance. **(B)** Naturally occurring epialleles have given phenotypic diversity in crops. The state of the OsSPL14 epiallele leads to differences in grain yield between rice varieties (upper). Cultivated cotton has acquired a trait of photoperiodic flowering by epigenetic change on COL2D during the domestication process (lower). **(C)** Paramutation of the b1 locus is established by the trans-acting epigenetic effect from a paramutagenic allele (B’) to a paramutable allele (B-I) and contributes to the phenotypic change in their progenies.

The strong indication that epigenetics is involved in heritable phenotypic change is shown by studies using epigenetic recombinant inbred lines (epi-RILs). Epi-RILs were generated by crossing between wild type and hypomethylated mutants, followed by repeated self-pollination of individual plants creating homozygosity of epigenetic states of each allele (Johannes et al., 2009; Reinders et al., 2009). Epi-RIL populations vary in DNA methylation states with minor DNA sequence variation. Phenotypic variation was identified in epi-RIL populations including some heritable traits such as flowering time and plant height (**Figure 2A**; Johannes et al., 2009; Zhang et al., 2013). Phenotypic variation was also found in quantitative traits, suggesting that epigenetic variation is involved in creating heritable phenotypic variation in quantitative traits (Johannes et al., 2009; Reinders et al., 2009; Cortijo et al., 2014). Epigenetic markers, which can detect the heritable differences in DNA methylation, can be applied to construct linkage maps and to identify epigenetic quantitative trait loci (QTLs) such as flowering time, primary root length, and clubroot resistance (**Figure 2A**; Cortijo et al., 2014; Liégard et al., 2019). The magnitude and stability of heritable phenotypic variation in epi-RILs is similar to that in RILs derived from two different accessions and natural accessions (Zhang et al., 2018b). The findings from epi-RILs suggest that epigenetic variation may also be involved in the phenotypic variation of agriculturally important traits. Furthermore, *A. thaliana* F_1_ plants generated by crossing between epi-RIL and wild type parents showed enhanced vegetative growth (Dapp et al., 2015; Lauss et al., 2018). DNA methylation repatterning caused by *msh1* resulted in enhanced growth in the F_4_ generation of crosses between *msh1* and wild type in *A. thaliana* (Virdi et al., 2015), and a similar phenomenon was observed in sorghum (Ketumile et al., 2022). These findings suggest epigenetic variation can potentially become sources for breeding high yielding crops.

## Crop phenotypes regulated by epigenetics

### Epigenetic marks related to plant phenotypes

Epigenetic states vary between plant organs and tissues, and this variation is related to specific-cell fate, normal plant growth and development. Some changes in the epigenetic state have been associated with developmental differences (**Table 1**) . For example, in *A. thaliana*, hypermethylation in the CHH methylation pattern is observed in columella relative to other root cell types, perhaps reinforcing silencing in neighboring stem cells (Kawakatsu et al., 2016b). CHH methylation changes dynamically during embryogenesis and germination, and this change of DNA methylation may affect seed dormancy (Kawakatsu et al., 2017). In the shoot apical meristem of both *A. thaliana* and rice, increasing CHG and CHH methylation levels are observed after phase transition from the vegetative to reproductive phase, and this change contributes to genome protection from harmful TEs in stem cells (Gutzat et al., 2020; Higo et al., 2020). A recent study has reported that significant differences of DNA methylation in the CHH context in several tissues can be attributed to the specific expression of *CLASSY* (*CLSY*) genes, which encode chromatin remodelers (Zhou et al., 2022). These results suggest that DNA methylation is altered during development, with specific patterns being required for normal development and for regulation of complex phenotypes, including agricultural traits.

**Table 1:** Summary of the epigenetic mechanism leading to various phenotypes in different plant species.

Species	Target gene or loci	Target phenotype	Epigenetic mechanism	Reference
***Arabidopsis thaliana***	*FWA*	Late flowering	Hypomethylation in the promoter region leading to higher expression	Soppe et al., 2000
	*DWF4*	Lack of elongation in shoots and petioles	Insertion of CACTA family transposon that is activated by hypomethylation	Miura et al., 2001
	*BNS*	Short, compact inflorescence, reduced plant height	Hypermethylation in the entire gene	Saze and Kakutani, 2007
	*FASCIATA1*	Abnormal flower development	Insertion of *gypsy* class retrotransposon that is activated by hypomethylation	Tsukahara et al., 2009
	QTLs	Enhanced growth	*De novo* epigenetic variation resulting from the use of *MSH1* silencing line	Virdi et al., 2015
	QTLs	Flowering time, plant height, fruit number, biomass, root:shoot ratio	Variation of DNA methylation resulting from the use of epigenetic inbred lines	Johannes et al., 2009; Reinders et al., 2009;
**Canola**	QTLs	Variation of energy use efficiency (EUE)	Epigenetic variation within the population	Hauben et al., 2009
**Cotton**	COL2D	Photoperiodicity	Hypomethylation on the 5’ region	Song et al., 2017
**Linaria vulgaris**	*Lcyc*	Radial symmetry (peloric) flower	DNA methylated and transcriptionally silent	Cubas et al., 1999
**Maize**	B1, R1, Pl1, P	Anthocyanin pathway pigmentation of various shoot tissues	Paramutation	Hollick, 2017
	low phytic acid 1	Reduced seed phytic acid, elevated seed inorganic P	Paramutation	Pilu et al., 2009
**Melon**	CmWIP1	Sex deteminaton	Hypermethylation on promotor leading formation of female flower	Martin et al., 2009
**Oil palm**	Karma	Mantled trait from somaclonal variant	Hypomethylation on karma	Ong-Abdullah et al., 2015
**Orange**	ripening-related genes	Ripening process	Hypermethylation during fruit-repening	Huang et al., 2019
**Persimmon**	MeGI	Sex deteminaton	Hypermethylation on the promoter region leading male flower, hypomethylation leads female flower	Akagi et al., 2016
**Rice**	QTLs	Energy use efficiency (EUE), enhanced seed yield	Epigenetic variation within the population	Schmidt et al., 2018
	RIZBZ1, RPBF	Aleurone layer formation	Demethylation by OsROS1a during grain maturation	Liu et al., 2018
	D1	Dwarfing	Hypermethylation and repressive histone mark on the promoter region	Miura et al., 2009
	OsFIE1	Dwarfing, flower developement	Hypomethylation and H3K9me2 depleation on the 5’ region	Zhang et al., 2012
	RAV6	Leaf angle	Hypomethylation on the promoter region	Zhang et al., 2015
	OsAK1	Chlorophyll formation	Hypermethylation on the promoter region	Wei et al., 2017
	OsSPL14	Grain yield	Hypomethylation on upstream 2.6-kb region	Miura et al., 2010
**Sorghum**	QTLs	Grain yield, tiller number, plant height, flowering time	*De novo* epigenetic variation resulting from the use of *MSH1* silencing line	Ketumile et al., 2022
**Soybean**	QTLs	Reduced growth rate, male sterility, enhanced branching and altered leaf and floral morphology	*De novo* epigenetic variation resulting from the use of *MSH1* silencing line	Raju et al., 2018
**Strawberry**	ripening-related genes	Ripening process	Demethylation during fruit-repening	Cheng et al., 2018
**Tomato**	*SLTAB2*	*SULFUREA*	Paramutation	Gouil et al., 2016
	QTLs	Leaf morphology, variegation, dwarfing, male sterility, flower development, and flower timing	*De novo* epigenetic variation resulting from the use of *MSH1* silencing line	Yang et al., 2015
	*LeSPL-CNR*	Abnormal ripening, colorless fruit	Silencing of expression by increased DNA methylaiton in the promoter region	Manning et al., 2006
	*CNR, VTE3*	Ripening process	Demethylation by SlDML2 during fruit-repening	Liu et al., 2018

A prominent case of an epigenetic trait important to a crop is the role of *Karma* in the “mantled trait” in oil palm (Ong-Abdullah et al., 2015). The mantled trait is a somaclonal variant arising from tissue culture that greatly reduces yield and has impeded efforts to clone elite hybrids for use in oil production. Ong-Abdullah et al. (2015) demonstrated that this trait resulted from hypomethylation of a *Karma* transposon insertion in the homeotic gene *DEFICIENS*. Hypermethylation of this insertion results in normal fruit set and high yields. Understanding the epigenetic basis of this trait will allow the oil palm industry to predict and cull mantling at an early stage of production, facilitating production of high-performing clones.

The fruit ripening process in tomato is controlled by DNA methylation. During fruit development, the expression of the DNA demethylase SlDML2 increases, and results in reduced global DNA methylation (Liu et al., 2015; Lang et al., 2017). The demethylation activates various ripening-related genes such as *CNR* and *Vitamin E 3* (*VTE3*) genes (Manning et al., 2006; Zhong et al., 2013; Quadrana et al., 2014). These genes are naturally hypermethylated in their promoter regions in immature fruit and non-fruit tissues (Zhong et al., 2013; Liu et al., 2015; Lang et al., 2017). Similarly, comprehensive demethylation is observed together with a reduction of RdDM activity during ripening of strawberry fruit (Cheng et al., 2018). In contrast, a fruit ripening process of sweet orange shows an increase in global DNA methylation levels caused by decreasing expression of the DNA demethylase gene (Huang et al., 2019). In rice, global DNA demethylation is also involved in controlling endosperm development. Two transcription factors, *rice seed b-Zipper* (*RISBZ1*) and *rice prolamin box binding factor* (*RPBF*) genes, repress aleurone formation (Kawakatsu et al., 2009). Rice DNA demethylase OsROS1a removes DNA methylation on the promoter regions, activating them during endosperm development, and restricting the aleurone layer. In contrast, a weak mutation of *OsROS1a* causes DNA methylation levels to be maintained in the *RISBZ1* and *RPBF* genes, increasing the number of aleurone cell layers and improving nutritional value of rice grains (Liu et al., 2018).

Sex determination in plants leads to the formation of unisexual flowers, with either pistils or stamens, enhances outcrossing and increases genetic diversity. In melon and some persimmon species, sexual forms are regulated by DNA methylation (Martin et al., 2009; Akagi et al., 2016). In melon (*Cucumis melo* L.), the expression of the *1-aminocyclopropane-1-carboxylic synthase* (*CmACS-7*) gene encodes an ethylene biosynthesis enzyme that represses stamen development in female flowers. In male flowers, wound inducible protein 1 (CmWIP1), a zinc-finger transcription factor, indirectly represses the expression of *CmACS-7* and, consequently, aborts carpel development and results in the development of unisexual male flowers. In addition, a DNA methylation change in the DNA transposon inserted into the *CmWIP1* promotor causes *CmWIP1* silencing, and the conversion from male to female flowers (Martin et al., 2009). In diploid persimmon (*Diospyros lotus* L.), an individual plant has either male or female flowers, and a pair of genes encoding the homeodomain transcription factors, *Male Growth Inhibitor* (*MeGI*) and the Y-chromosome encoded pseudogene *Oppressor of MeGI* (*OGI*), governs sex determination (Akagi et al., 2014). MeGI represses anther development, and *OGI* produces 21-nt siRNAs that silences *MeGI* post-transcriptionally, leading to stamen development. In contrast, hexaploid persimmon, *D. kaki*, is a monoecious species in which an individual plant has both male and female flowers. Flexible regulation of *MeGI* expression determines the formation of male or female flowers, resulting from *OGI* silencing by retrotransposon insertion in promoter regions. *MeGI* expression is repressed in male flowers by DNA methylation at the *MeGI* locus, and demethylation at this locus forms female flowers (Akagi et al., 2016).

Some repressive histone marks also play a critical role in suppressing gene expression and regulating plant development. One example is H3K27me3, which is maintained by the widely conserved Polycomb repressive complex 2 (PRC2). Although DNA methylation levels often differ between lines within a species, H3K27me3 patterns are mostly conserved between different lines or varieties in the same species (He et al., 2010; Makarevitch et al., 2013; Akter et al., 2019). However, the distribution of H3K27me3 is very different between tissues (Makarevitch et al., 2013), suggesting that PRC2 and H3K27me3 play an essential role in determining cell fate and normal plant development. In *A. thaliana*, PRC2 forms three complexes that control distinct developmental transitions (Mozgova et al., 2015); EMBRYONIC FLOWER (EMF) and VERNALIZATION (VRN) class PRC2 proteins controls sporophyte development and the developmental transition of flowering, while FERTILIZATION-INDEPENDENT SEED (FIS) class PRC2 regulates female gametophyte and early seed formation *via* the regulation of genomic imprinting (described in the next section). Although the characteristics of PRC2 complexes have not been fully understood in crops, some rice studies reported the roles of PRC2 (Tonosaki and Kinoshita, 2015). The rice flowering transition responds to day-length and is also regulated by PRC2 -containing OsEMF2b with a chromatin remodeling factor VIN3-LIKE 2 (OsVIL2) (Luo et al., 2009; Yang et al., 2013). Two recent studies have revealed that OsEMF2a-containing PRC2 regulates female gametogenesis and early endosperm development, similar to the *A. thaliana* FIS-class PRC2 (Cheng et al., 2021; Tonosaki et al., 2021), although FIS-class PRC2 is only conserved in Brassicaceae species (Luo et al., 2009). Phenotypes and the contributing epigenetic mechanism for each species have been summarized in **Table 1**. In crop plants, developmental processes influence crop yield and efficiency of breeding; further investigation of the epigenetic mechanisms of plant phenotypes affecting crop yield and efficiency of breeding is necessary.

### Naturally occurring epialleles for phenotypic diversity

Phenotypic diversity between plants can arise from either genetic mutations or stable non-genetic changes in the form of epimutations. Although reports of spontaneous epialleles that confer phenotypes are rare, some studies have demonstrated that naturally occurring epialleles have been involved in phenotypes in various crops (Samantara et al., 2021). For example, naturally occurring epialleles of the *VTE3* gene in tomato, mediated by differential methylation of a SINE retrotransposon inserted in the promoter, determine nutritionally important vitamin E levels in tomato fruit (Quadrana et al., 2014).

Several epialleles associated with agronomic traits have been found in rice. An epiallele of the *DWARF1* (*D1*) gene encoding the GTP-binding protein, *Epi-d1*, shows a metastable dwarf phenotype (Miura et al., 2009). This phenotype is caused by hypermethylation and association with a repressive histone mark on the promoter region of the *D1* gene, but there is no change in DNA sequence. *Epi-df* also shows a dwarf phenotype with various floral abnormalities inherited in a dominant manner (Zhang et al., 2012). In *Epi-df*, DNA methylation and H3K9me2 marks are reduced on the 5’ region of *FERTILIZATION-INDEPENDENT ENDOSPERM1* (*FIE1*) gene encoding a component of the rice PRC2. This change causes ectopic expression of *FIE1*, decreasing H3K27me3 and activating the PRC2-mediated genes. Likewise, change in DNA methylation levels on other rice epialleles, *Epi-rav6* and *Epi-ak1*, alters leaf angle by modulating brassinosteroid homeostasis, and photosynthetic capacity, respectively (Zhang et al., 2015; Wei et al., 2017). These naturally occurring epialleles are often stably inherited and independent of genetic variation and may be valuable material for altering agronomic traits for crop improvement.

Stable epiallele phenotypes observed in the above examples and *A. thaliana* epi-RILs may be rare, suggesting that latent epialleles are lost during sexual reproduction. The re-programming mechanism during gametogenesis is thought to rapidly reset the epigenetic states from the vegetative development phase (Tao et al., 2017). This may impede inheritance of most naturally occurring epimutations from the parental plant. In addition, the state of an epiallele is occasionally bidirectionally mutable, from active to repressed and from repressed to active, resulting in revertant phenotypes in a few progeny (Manning et al., 2006; Miura et al., 2009; Zhang et al., 2012; Zhang et al., 2015). DNA methylation can be inherited to the progeny, but this may depend on environmental conditions such as biotic and abiotic stresses (Tricker, 2015). The reversion of an epiallele and variability of DNA methylation carry the risk of losing the selected phenotype or not being able to fix the epiallele through breeding programs. However, vegetatively propagated plants without any reproductive phase may allow reliable and stably maintained inheritance of epialleles that are useful for breeding programs.

Some studies have indeed reported that epialleles related to important traits have been stably maintained during improvement and domestication (**Figure 2B**). The rice epiallele, *OsSPL14*, is involved in rice grain yield. Higher expression of *OsSPL14* promotes panicle branching and the number of grains per panicle, but this expression is suppressed in a typical japonica rice variety by high DNA methylation in the upstream region of *OsSPL14* (Miura et al., 2010). Since expression of *OsSPL14* and DNA methylation levels of the locus are associated with rice grain yield, this epimutation may determine the yield phenotype between different rice varieties. In cotton, comprehensive epigenomic analysis between domesticated cottons and their relatives found that some differentially methylated genes contribute to domestication traits, including flowering time and seed dormancy (Song et al., 2017). The photoperiodicity-related gene, *CONSTANS-LIKE 2D* (*COL2D*), is highly methylated in wild cotton; however, *COL2D* in domesticated cottons is hypomethylated and activated; perhaps resulting in photoperiod dependent flowering in domesticated cotton. Similarly, a large number of DMRs contribute phenotypic variation between maize and teosinte and show signals of selection during domestication (Xu et al., 2020b). These epimutations may have produced domesticated phenotypes in crops with epigenetic marks being maintained during domestication.

#### Paramutation

Naturally occurring epialleles can lead to the epigenetic phenomenon referred to as “paramutation” (Pilu, 2015). The non-Mendelian pattern of inheritance typical of paramutation was first observed in studies of the genetics of “rouges” in cultivated peas (*Pisum sativum* L.; Bateson and Pellew, 1915). and subsequently in studies of the inheritance of pigmentation phenotypes associated with the anthocyanin pathways in maize (Brink, 1956), and in studies of inheritance at the *sulfurea* locus in tomato (Hagemann, 1958; Hagemann and Berg, 1978). The power of the anthocyanin pigment pathway as a model for genetic studies in maize, perhaps combined with the importance of maize as a major food crop, has led to advances in understanding the biology and molecular genetics of paramutation in this species (Hollick, 2017; Springer and Schmitz, 2017). While the basic biology of paramutation is mostly similar across plant species there are important differences, as illustrated by differences between maize and tomato (Gouil et al., 2016). A fuller understanding of the similarities and differences between species will allow development of a more accurate general model of the biology of paramutation, than would studies of maize alone (Gouil et al., 2016). This remains an active field of research, with recent progress ranging from studies of the original model, the rogue phenotype in pea (Pereira and Leitão, 2021), to studies in tomato (Martinho et al., 2022), *A. thaliana* (Bente et al., 2021), and continuing studies in maize (Deans et al., 2020). The studies in cultivated species like pea, tomato, and maize illustrate that paramutation is not just an interesting epigenetic phenomenon but has substantial economic and cultural importance. For example, paramutation occurs at the maize *low phytic acid* 1 gene, a gene of some significance to the nutritional quality of seed crops when used either for animal feeds or human foods (Pilu et al., 2009).

In paramutation, a *trans*-acting regulation can convert a paramutable allele to a paramutagenic allele on the same locus (Chandler, 2010). A well-defined example is the maize *b1* locus that encodes a transcription factor involved in anthocyanin biosynthesis (Coe, 1959). The *B’* allele at the *b1* locus has a low expression level and produces light (or pale) pigmentation in the whole plant body, while the *Booster-Intense* (*B-I*) allele is highly transcribed and provides dark pigmentation. When the *B-I* allele is exposed to *B’* alleles, *B-I* is converted to *B’* and pigmentation changes from dark to light in all F_1_ plants (**Figure 2C**); this change in the state of the paramutable *B-I* allele is stably inherited even after the loss of the paramutagenic *B’* allele during segregation. The *B-I* and *B’* alleles are epialleles; both have identical DNA sequences, but there is a distinct difference in DNA methylation and chromatin structure at multiple tandem repeats that act as a long-range enhancer element (Stam et al., 2002a; Stam et al., 2002b; Louwers et al., 2009; Haring et al., 2010). However, studies of *sulfurea* paramutation in tomato illustrate that neither tandem repeats nor their function as a long-distance enhancer acting on a gene’s promoter are essential: *sulfurea* paramutation occurs *via* hypermethylation of the transcription start site of the target gene (*SLTB2)* and doesn’t involve tandem repeats (Gouil et al., 2016). What is common between paramutation at maize *b1* and tomato *SLTB2* is the role of the RdDM pathway.

Genetic analyses have identified several genes that are necessary for establishment and/or maintenance of the paramutation and involve the production of small RNAs and function of the RdDM pathway (Hollick, 2017). RdDM is causative for gene silencing by guided *de novo* DNA methylation *via* small RNAs (Matzke and Mosher, 2014). The tandem repeat region of *B-I* to *B’* alleles generates small RNAs, and overexpression of siRNAs derived from the tandem repeat can induce paramutation in *trans* (Arteaga-Vazquez et al., 2010), while the absence of the RdDM-induced trigger resulted in loss of paramutation (Arteaga-Vazquez et al., 2010). This result suggests that the RdDM pathway and small RNAs are important to mechanisms underlying paramutation but cannot fully explain these mechanisms. Paramutation-like phenomena in maize and other plant species appear to be controlled by similar epigenetic regulation involving the interaction of small RNAs (Pilu, 2015; Hollick, 2017). The *trans*-acting epigenetic effects of paramutation can be stably and transgenerationally inherited and are expected to contribute to the generation of the phenotypic diversity without any genomic change. However, inheritance of paramutation deviates from Mendelian segregation and may make the selection of important traits difficult.

Advances in the study of the molecular biology of paramutation in maize was facilitated by the isolation of stable, genetic mutations of genes that are functionally critical to this phenomenon, yet the genetics of paramutation is not fully elucidated. While the first case of paramutation, the rouge phenotypes in pea, was documented over 100 years ago, no such genetic studies had been reported until the recent work of Pereira and Leitão (2021). These authors developed a “non-rogue” pea line harboring a “neutral” (in terms of paramutation) allele, analogous to similar mutations in maize, isolated from a chemically-mutagenized population. Interestingly, the penetrance of the non-rouge mutation was not complete and paramutation could still be observed in some exceptional cases, indicating that the paramutation system in pea may differ in part from that in maize. Additional examples of continuing genetics progress also demonstrated inter-species functional conservation (Deans et al., 2020; Martinho et al., 2022). Deans et al. (2020) demonstrated that the maize ortholog of the *A. thaliana* PICKLE-like helicase DNA-binding 3 protein, CHD3, plays a role in the maintenance of the *purple plant* 1 (*pl*1) paramutant alleles, and also in supporting both normal somatic development and gametophyte function, *via* the maintenance of hypermethylated “off” states of given alleles. Furthermore, since they both function to maintain “off” states, CHDs and the RNA polymerase II catalytic subunit RPD1, which plays a role in generating the small RNAs important to paramutation, are mechanistically linked. Martinho et al. (2022) demonstrated that the *sulfurea* paramutation in the tomato is dependent on the function of the tomato ortholog of *A. thaliana*’s *KYP* and *CMT3*. Furthermore, this result indicates that models of paramutation should include CMT3/KYP and its role in changing the chromatin structure, in addition to mechanisms that involve small RNAs and the RdDM pathways, and that these models should accommodate both locus-specific and species-specific mechanisms.

While paramutation was initially studied at a limited number of loci in maize, subsequent studies have found that it represents a more common, genome-wide phenomenon. A study of “expression quantitative trait loci” (e-QTLs) in maize found that 145 genes displayed patterns of non-Mendelian e-QTL inheritance that was paramutation-like: where all the segregating progeny from a cross had expression levels for a given gene similar to either one or the other of the two parents (Li et al., 2013). Genome-wide mapping of cytosine methylation in two maize inbreds revealed widespread paramutation-like “switches” guided by small RNAs (Regulski et al., 2013). Similar genome-wide, multi-locus paramutation-like phenomena have also been documented in *A. thaliana* (Greaves et al., 2014) and in tomato (Gouil and Baulcombe, 2018). Clearly paramutation-like non-Mendelian inheritance that is commonplace and genome-wide could greatly impact crop breeding efforts that are designed based on Mendelian principles of inheritance.

The epigenetic phenomena of inter- and transgenerational inheritance or memory of responses to stress or environmental change experienced by parents and transmitted to offspring (Section 5), has been documented to impact paramutation (Mikula, 1967; Bente et al., 2021). In a study of paramutation at the *R* locus in maize (Mikula, 1967), testcross scores reflecting paramutation were increased when plants used as males in testcrosses were grown in the greenhouse under a 12 hr light/12 hr dark photoperiod rather than a 24 hr light photoperiod. Since the differentially treated plants were used as males in the testcrosses to plants that were not grown under the different photoperiods, any effects must have been transmitted through the pollen. The paramutant phenotype was then observed in the next generation. Therefore, this inheritance could not be attributed to a maternal effect and thus is properly referred to as “transgenerational inheritance” rather than “intergenerational inheritance” (for an explanation of this important distinction, see Section 5). More recently, Bente et al. (2021) demonstrated that temperature differences during the growth of *A. thaliana* hybrids harboring a paramutable reporter transgene played a role in the degree and timing of its interaction with the paramutagenic allele.

#### Genomic imprinting

Genomic imprinting is an inherently epigenetic phenomenon that shows a parent-of-origin dependence and involves preferential expression from one parental allele. Imprinted genes are found predominantly in the endosperm tissue of angiosperms but are rare in embryos. A gene could be preferentially expressed from the maternal allele when it is classified as a maternally expressed gene (MEG) or from the paternal allele, a paternally expressed gene (PEG). Based on studies mostly in *A. thaliana*, preferential expression of imprinted genes is explained by strict epigenetic reprogramming during gametogenesis. In the central cell of a female gamete, the DNA methylation level is widely reduced by the action of DNA demethylase (Park et al., 2016). However, DNA methylation in a sperm cell is maintained and reinforced possibly *via* the siRNA pathway derived from the vegetative cell (Martinez et al., 2016; Kim et al., 2019). The different DNA methylation state between gametes leads to the maternal expression of MEGs. In contrast, H3K27me3 is reduced in sperm cells by erasers such as H3K27me3 demethylase but maintained in the central cell by the PRC2 (Mozgova and Hennig, 2015; Borg et al., 2020), resulting in the paternal expression of PEGs. In addition, a recent study has revealed that preferential expression in almost all PEGs is strengthened by multiple repressive regulation on the maternal allele (Moreno-Romero et al., 2019). These asymmetric epigenetic regulations between parental gamete are natural epialleles and establish genomic imprinting in the endosperm.

Numerous imprinted genes have been found in many plant species, including crop plants. Loss-of-function of imprinted genes can often result in crucial developmental defects, such as seed abortion (Luo et al., 2000; Tonosaki et al., 2021). The most accepted explanation for the biological significance of imprinted genes is the parental-conflict hypothesis for allocating limited maternal resources to offspring (Haig and Westoby, 1989). Several crop studies showed evidence that some imprinted genes function to control nutrient allocation. In maize, *Meg1* is a maternally imprinted gene and involves establishing and differentiating endosperm nutrient transfer tissue (Costa et al., 2012). Bi-allelic expression of *Meg1* causes more significant expansion of the transfer tissue and leads to providing disproportional maternal resources to the endosperm; the dosage regulation of imprinted genes is critical for a balanced distribution of maternal nutrients to seeds (Costa et al., 2012). In maize, some MEGs are also involved in nutrient accumulation and allocation in the endosperm (Xin et al., 2013). In rice, the knockout of *OsEMF2a*, a MEG encoding a component of rice PRC2, induces autonomous endosperm development without fertilization (Tonosaki et al., 2021). Rice autonomous endosperm can produce storage compounds, starch granules and protein bodies, while endosperm cell structure is not acquired (Tonosaki et al., 2021). This result suggests that an imprinted *OsEMF2a* regulates the downstream pathway to determine the nutrient allocation to the endosperm. In addition, a recent study has reported that a maternally imprinted long-noncoding RNA, *MISSEN*, prevents H3K27me3 modification after pollination that is essential and quantitatively regulates the size of the rice endosperm (Zhou et al., 2021). Although endosperm is a tissue that is eventually terminated and the epigenetic state is not transmitted to the progeny, epigenetic variation leads to variation of genomic imprinting and generates a novel gene expression pattern in seeds (Pignatta et al., 2014). It is possible that the crop grain quality and quantity can be improved using epialleles or by modification of the epigenetic state.

Imprinting of genes has a remarkable role in the hybridization barrier, preventing the introgression of genes from other species during interspecific and interploidy crossing. The fusion of the two genomes from different species or disturbing the parental genomic ratio in the endosperm leads to a parent-of-origin seed abortion phenotype (Kradolfer et al., 2013; Wolff et al., 2015; Tonosaki et al., 2016). The seed abortion phenotypes are associated with mis-regulation of expression of imprinted genes and seed viability can be recovered by rescuing the expression of imprinted genes (Kradolfer et al., 2013; Schatlowski et al., 2014; Wolff et al., 2015; Erdmann et al., 2017; Huang et al., 2017; Martinez et al., 2018; Wang et al., 2018). These results indicate that the preferential expression pattern of imprinted genes is critical for endosperm development. Because imprinted genes are sensitive to changes in parental genome dosage, the adjustment of genome dosage between parental species leads to recovering preferential expression pattern of imprinted genes and overcomes the hybridization barrier in interspecific crosses (Lafon-Placette et al., 2017; Tonosaki et al., 2018).

## Technologies to modify the epigenome

More agricultural traits are being found to be regulated by epigenetics, and fast integration of the epialleles into the desired cultivar is important for breeding. Natural epigenome modifications have been acquired through evolution and traditional breeding based on trait and genetic characterization. Artificial induction of epigenome modification is expected to increase the phenotypic diversity including agronomically useful traits. Broadly, there are two methods for epigenome modification, random and targeted. “Random” epigenome modification induces epigenome changes randomly into the genome, whereas “targeted” allows epigenome modification of specific regions of the genome.

### Random epigenome modification

#### Tissue culture

**Figure 3:**
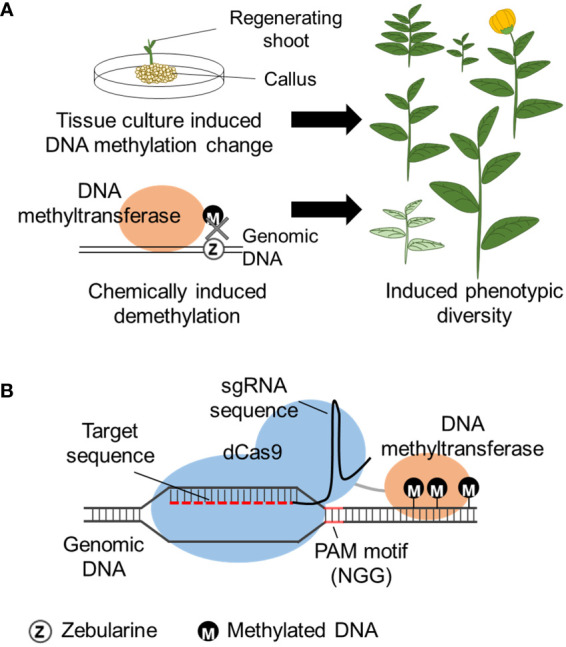
Methods to induce epigenetic changes. **(A)** DNA methylation is altered during tissue culture, or chemical (zebularine) induced random DNA demethylation by inhibition of DNA methylation, leading to gene expression change and induced phenotypic diversity. **(B)** CRISPR/dCas9 induced DNA methylation by fusion of DNA methyltransferase to the dCas9 protein. The dCas9 fusion protein is directed to the homologous regions (target sequence) of the designed sgRNA and induces methylation to the flanking DNA region. The target sequence requires a PAM motif (N can be A, C, G or T) within the sgRNA design.

Tissue culture is a commonly used technique to establish and regenerate plants from tissue explants by aseptically placing the tissue on plant growth medium. The initial medium contains phytohormones adjusted to induce undifferentiated cells growth (callus). The explant is then placed on media to induce shoot and root formation. The phytohormone concentration required for each developmental stage can depend on the plant species and regeneration of plants from callus can take weeks or even months depending on the plant species. Regenerated plants from the same tissue are clones harboring the same genome, but has phenotypic variation, named somaclonal variation. Somaclonal variation is known to be caused by both genetic and epigenetic changes of the genome that occur during the tissue culture. DNA methylation levels are reported to increase with the 2,4-dichlorophenoxyacetic acid (2,4-D) concentration that is used for callus induction and increases with kinetin concentration that induces shoot formation (Azizi et al., 2020). Tissue culture induced heritable DNA hypomethylation has been observed in oil palm (Ong-Abdullah et al., 2015), maize (Stelpflug et al., 2014), rice (Stroud et al., 2013), and *A. thaliana* (Tanurdzic et al., 2008). Establishment of plant regeneration by optimizing tissue culture conditions can be challenging, but the use of this technique can induce phenotypic varieties that may be useful for breeding (**Figure 3A**), especially applicable for plants that already have an established tissue culture system.

#### Chemical induced DNA methylation changes

DNA methylation inhibitors have been frequently used to modify the natural DNA methylation that occurs within the genome. Namely, 5’-aza-2’-deoxycytidine (5-azadC) and zebularine are cytosine analogs that are randomly incorporated into the genome during DNA replication, replacing cytosine. In the case of 5-azadC, the azacytosine-guanidine dinucleotides are recognized and bound by DNA methyltransferase but cannot accept the transfer of the methyl group and become trapped as an adduct ultimately leading to the inhibition of DNA methylation (**Figure 3A**). Zebularine has a similar mechanism involving the formation of a covalent adduct with DNA methyltransferase (Cheng et al., 2003; Champion et al., 2010). Use of zebularine on wheat (*Triticum aestium* L.) created a line with a heritable increase of spikelet number possibly caused by epigenetic modification (Finnegan et al., 2018). However, care is needed for the interpretation of 5-azadC and zebularine epimutagenesis because it is known to also induce genetic mutations (Jackson-Grusby et al., 1997). As a random epigenome modifier, RNA polymerase II (PolII) inhibitors also can function as DNA methylation inhibitors (Thieme et al., 2017) because PolII is required for RdDM establishment of some loci (Gao et al., 2010). Seedlings treated with a PolII inhibitor, alpha-amanitin, decreased PolII activity and led to global DNA hypomethylation and phenotypic variability (Thieme et al., 2017). The effects of the chemicals are usually transient and the epigenome of many loci is restored once the chemical is removed (Baubec et al., 2009).

DNA hypomethylation can result in the activation of mobile TEs that can translocate within the genome by a cut-paste (DNA transposons) or copy-paste (retrotransposons). The transposing TEs may land in a position that will disrupt gene function, leading to heritable genetic changes. These mutations can induce useful agronomical traits. Epigenetic breeding can result in genetic changes and is important to understand the underlying mechanism of the phenotypic change in order to consider the appropriate breeding strategy for crop improvement. For this, the combination of efficient phenotypic, genetic, and epigenetic screening systems may be required to identify useful traits from a large-scale random epigenome modification.

### Targeted epigenome modification

#### RNA interference

RNAi was developed in the late 90’s as a means to regulate specific gene expression *via* epigenetic modifications. RNAi can be induced by the expression of transgenes designed to form an inverted repeat separated by a spacer that folds into a hairpin RNA (hpRNA) structure (Baulcombe, 2004). This hpRNA is then processed into siRNAs that mediate post transcriptional transgene silencing (PTGS) and subsequently transcriptional gene silencing (TGS) by the RdDM pathway. Similarly, virus induced gene silencing (VIGS) has also been used to induce PTGS and RdDM in plants (Abrahamian et al., 2020). These methods can efficiently trigger PTGS but not necessarily TGS (Eamens et al., 2008). The exact mechanism is not known, but there seems to be a mechanism to protect some endogenous genes from TGS. However, non-polyadenylated or hpRNA transgenes inserted into an intron show efficient RdDM and TGS (Mette et al., 1999; Dalakouras et al., 2009). The heritable epigenetic state may depend on the target gene but may be made more efficient by designing the RNAi to intronic regions. Further understanding of the exact mechanism of epigenome heritability is anticipated to allow more efficient and flexible design of RNAi for any epialleles of interest.

#### TALE and CRISPR/Cas9

An alternative method for epigenome modification is *via* the use of genome editing enzymes. Transcription activator-like effectors (TALEs) and Clustered regularly interspaced short palindromic repeats/CRISPR-associated protein 9 (CRISPR/Cas9) are enzymes that have been used for targeted epigenome editing. TALEs are proteins that are naturally occurring transcriptional activators secreted by the plant pathogen *Xanthomonas* spp. Fusing a DNA methylation or demethylating enzyme such as DNA methyltransferase 3 alpha (DNMT3A) or ten-eleven translocation 1 (TET1) to TALE has been used for DNA methylation modification in mammalian cells (Lo et al., 2017).

Cas9 is a DNA endonuclease that associates with a single-guide RNA (sgRNA). sgRNA consists of a 20 nucleotide sgRNA recognition sequence containing a protospacer adjacent motif (PAM) that has 5’-NGG-3’ sequence and a scaffold sequence for Cas-binding. Cas9/sgRNA complex is directed to bind to the recognition sequence on the genomic DNA. For epigenome editing, a dead Cas9 (dCas9) is used so the Cas9 will still bind to the target DNA but will not cleave the site. Similar to TALEs, an epigenomic modifying enzyme can be fused to dCas9 and direct the epigenome modification in the target region (**Figure 3B**). Both enzymes have lower binding efficiency on heterochromatic regions, however, TALEs have recently been reported to have higher binding efficiency than Cas9 for genome editing (Jain et al., 2021), and this can also be expected for epigenome editing [e.g., for DNA demethylation (Moradpour and Abdulah, 2020)].

### Implementation in breeding

One of the important considerations for using artificially induced epigenome modification is the legal or political regulation concerning the safety of the consumers and environment. Genetically modified organisms (GMOs) have been regulated in many countries to limit their use to within a closed environment to prevent the release of any transgenic plant material (including pollen) to an open environment. For releasing transgenic plants for commercial use, a strict and often expensive risk assessment is often required, which may outweigh the benefit of transgenic plants.

Conventional mutagenesis using ionizing/non-ionizing irradiation and chemicals have created useful traits for various crops. These techniques can induce structural changes of the genome including deletions, insertions, and chromosomal rearrangements (Hase et al., 2020; Ma et al., 2021). These mutagenized plants were generated long before GMO regulations existed and have been used by breeders for crop improvement. Random epigenome mutagenesis by tissue culture or chemicals, which does not introduce transgenes, are also not under GMO regulation and could be used for breeding purposes in a similar way to genetically mutated varieties.

On the other hand, RNAi, TALE, and CRISPR/Cas9 technologies all require the introduction of transgenes to induce targeted epigenome modification. Therefore, any of these plants would be under GMO regulation, and would be difficult to use for any breeding that requires the release of the plant to an open environment. Recently some countries such as United States, Argentina, Australia, and Brazil have indicated that if no foreign DNA is present in the variety, it would not be regulated as a GMO and would be regulated equivalently to conventional crops (Smyth, 2020). Taking this into consideration, it may be possible to cross the epigenetically modified crops to non-transgenic elite varieties to segregate out the transgenes and select for desired lines assisted by epigenetic markers.

Alternatively, transient expression or external application of epigenome modifying transgene or proteins may be delivered to the plant for epigenome modification without transgene integration into the genome. Cell penetrating peptide is one such method that allows DNA/RNA/proteins bound to a designed peptide to transfer across the cell wall/membrane *via* endocytosis (Numata et al., 2018; Oikawa et al., 2021). Transfection of preassembled Cas9/sgRNA complex has been demonstrated (Woo et al., 2015; Murovec et al., 2018), and cell penetrating peptide has efficiently delivered proteins into plant cells (Bilichak et al., 2015; Terada et al., 2020). The Cas9/sgRNA delivery by cell penetrating peptides is expected to result in highly efficient genome editing.

**Figure 4:**
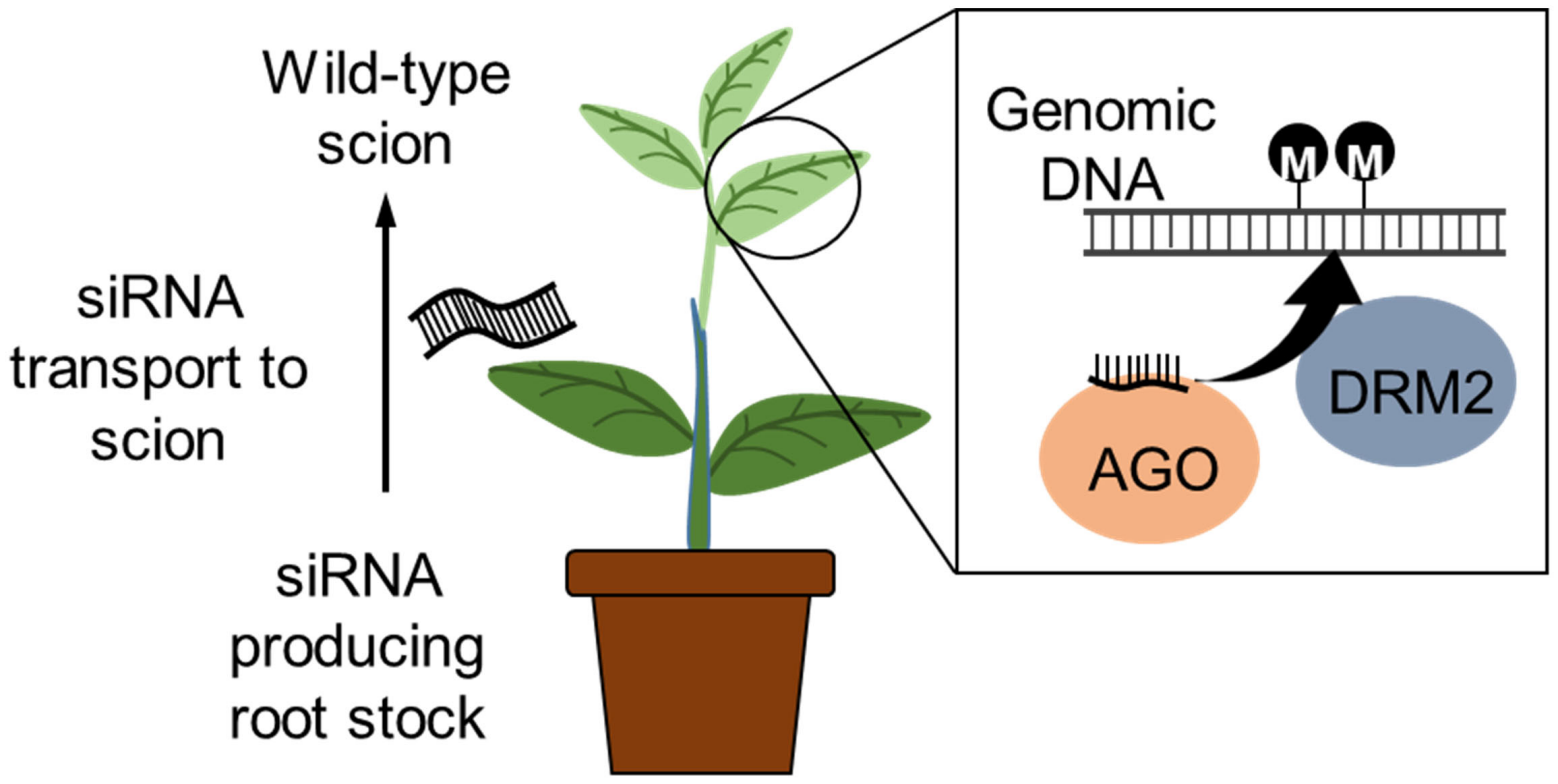
Inducing epigenetic changes by grafting. RNAi construct expressed in the rootstock produces siRNAs that induces DNA methylation. The siRNA is known to transport within the plant, and grafting a wild-type scion to a siRNA producing transgenic rootstock will transport the siRNA to the wild-type scion. The siRNA associates with the ARGONAUTE (AGO) protein and induces de novo DNA methylation via the RdDM pathway. The wild-type scion will be non-transgenic (free of transgene integration into the genome).

Similarly, grafting has also been reported to transport small RNAs from the scion towards the root stock *via* the plasmodesmata and sieve elements (Kasai et al., 2016). An siRNA producing tobacco (*Nicotiana benthamiana*) scion was grafted to a potato (*Solanum tuberosum*) rootstock and TGS induced *via* the RdDM pathway; the TGS was maintained in the progeny lacking the siRNAs. Rootstock-to-scion transfer of small RNAs conferring virus resistance in the scion of cherry tree was also demonstrated by grafting of a virus resistant rootstock variety. Grafting of lines that express RNAi or CRISPR/Cas9 transgenes for epigenome modification to an elite variety may allow more efficient epigenetic breeding without the need for finding a graft compatible line with the desired epiallele (**Figure 4**). Furthermore, the identification and application of epi-markers (that relates to agronomic trait and epigenetics changes) have potential value for efficient breeding (Turcotte et al., 2022), and these technologies will also be useful for screening epigenome edited crops.

## Can epigenome-mediated transgenerational stress memory enhance crop performance and productivity?

During development, plants display a wide array of adaptive mechanisms in response to changes and challenges in their environment, including biotic or abiotic stresses. These responses can result in intragenerational adaptive phenotypic change (Mozgova et al., 2019; Bhar et al., 2021). For example, multiple intragenerational exposures of *A. thaliana* to water stress “train” plants to better respond to subsequent exposure to that stress (Ding et al., 2012). In another example, analysis of the methylome revealed a central role for intragenerational epigenetic change in the adaptive response of *A. thaliana* to phosphate starvation (Yong-Villalobos et al., 2015). It is important to note that what constitutes an adaptive change is context dependent (Skirycz et al., 2011). These authors demonstrated that responses allowing survival of *A. thaliana* under extreme drought were not of benefit to plant growth and productivity under more moderate drought conditions. Thus, responses to severe stress may not be of benefit under the more moderate conditions typical of standard crop production environments. This can account for the low level of success in translation of findings of many basic studies of stress response to the applied field of crop production (Skirycz et al., 2011).

**Figure 5:**
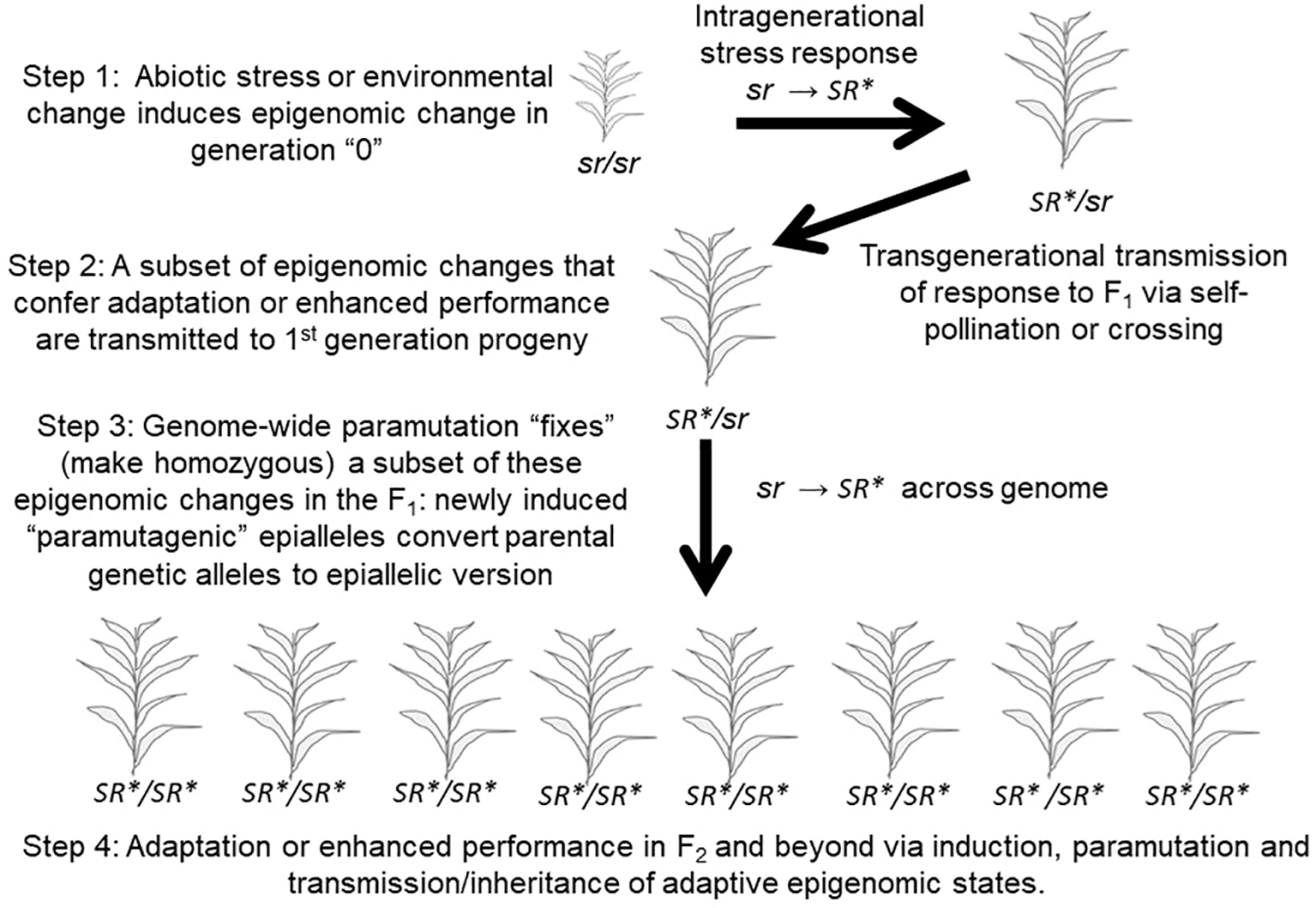
A Pseudo-Lamarckian Process that combines transgenerational adaptation to stress combined with paramutation. This process may rapidly fix (make homozygous in all progeny) a stress-induced, adaptive epigenomic change. “sr”: the epigenomic state of an individual prior to a given stress. “SR*”: the epigenomic state of an individual following response to a given stress.

Some studies have now demonstrated that plants are able to transmit a portion of this intragenerational response to biotic and abiotic stress, or to environmental change, to their offspring, *via* the epigenome-mediated phenomenon referred to as “transgenerational inheritance” (**Figure 5** top; Herman and Sultan, 2011; Mozgova et al., 2019). To accurately refer to a case of epigenetic inheritance as transgenerational, the inheritance of the epigenetic change in response to the stress or environmental change experienced by a parent should be observed in second- and/or subsequent generations in the absence of the inducing stress and in progeny whose embryonic and subsequent development occurred in the absence of that stress (Heard and Martienssen, 2014). If the epigenetic response is only observed in the first generation of offspring that began their development as seed developing in the maternal plant then that could simply be a type of maternal or parental effect and should be referred to as an “intergenerational” effect. A common maternal effect is “provisioning” of the developing seed by the maternal plant with important nutrients and other compounds (Herman and Sultan, 2016). This distinction is important in studies of animal and human epigenetic inheritance and is adhered to in that field (Heard and Martienssen, 2014). We will adhere to it here even though nearly all studies in plant systems use the term “transgenerational” broadly to refer to both intergenerational and transgenerational inheritance. In a transgenerational parallel to the intragenerational “training” described by Ding et al. (2012), a further potentially valuable distinction should be made. If the magnitude of the response or epigenetic change is substantially greater in magnitude in the 2^nd^ generation or subsequent progeny than that observed in progeny of unstressed parents when exposed to the same stress, then that could properly be referred to as a transgenerational memory effect; a case of “transgenerational training” that is often referred to as “transgenerational priming” (Holeski et al., 2012).

As an example of a transgenerational biotic stress memory that imparts adaptation, exposure of *A. thaliana* plants to caterpillar herbivory resulted in an epigenome-mediated jasmonic acid-dependent defense that persisted for two subsequent generations in the absence of the biotic stress (Rasmann et al., 2012), and thus was transgenerational as defined by Heard and Martienssen (2014). However, the adaptation to herbivory did not persist into the third generation. In fact, transgenerational adaptation and priming to both herbivory and pathogen attack is taxonomically widespread across plant species (Holeski et al., 2012). In some cases, pathogen attack can result in transgenerational resistance to both the primary pathogen and other pathogens (reviewed in Holeski et al., 2012). Herbivory on the wild radish (*Raphanus sativus* L.) by the cabbage butterfly (*Pieris rapae* L.) resulted in transgenerational priming of the adaptive response of increased trichome density; a greater-in-magnitude response to herbivory was observed in progeny whose parent was stressed than in those whose parent was not stressed (Sobral et al., 2021).

As an example of a transgenerational abiotic stress memory that imparts tolerance, limiting nitrogen supply to rice plants induced epigenomic change that was heritable through two progeny generations in the absence of the inducing stress and that conditioned enhanced tolerance to the same stress (Kou et al., 2011). Climate change poses a critically important challenge to world crop production, and water scarcity is at the forefront of that risk (World Economic Forum, 2015). A study of transgenerational response to water deficit in the annual plant *Polygonum persicaria* demonstrated that the adaptation can be attributed to differential DNA methylation and not due to the maternal effect of provisioning (Herman and Sultan, 2016). In peanut production, parental exposure to water deficit resulted in an enhancement in field emergence in progeny for three subsequent generations (Racette et al., 2020). However, of the five peanut genotypes included in the study, enhanced field emergence was only observed for two in each of the second and third years of study, and these two differed in each year. A combination of water and heat stress in durum wheat seedlings resulted in an intragenerational effect, apparently mediated by differential micro-RNA expression, that imparted tolerance to nitrogen limitation in progeny of stressed parents (Liu et al., 2021). While these authors referred to the effect as transgenerational, only the first progeny generation was studied. Thus, while this effect might in-fact be truly transgenerational, it can only be defined as intragenerational, according to the distinction described above. Of note is that this represented the first case of “cross-stress” adaptation, in that the parents were exposed to water and heat stress and the adaptation was observed for nitrogen stress. Drought stress of the parental plants was reported to result in a positive, intergenerational effect on seedling growth in the F_1_ in oilseed rape (*B. napus*), although it was referred to as a transgenerational effect by the authors (Hatzig et al., 2018).

This brief review reveals that the phenomenon of transgenerational inheritance may typically be of limited generational endurance, quite variable and inconsistent across genotypes of the same species and between species, and often conflated with what is properly referred to as intergenerational effects. It may also depend greatly on the severity of the stress or environmental change or result from a combination of stresses rather than a single stress. “Mild” drought during the vegetative stage in *A. thaliana* generated intragenerational changes reflecting response to drought including changes in genomic DNA methylation patterns (Van Dooren et al., 2020). However, one generation in the absence of the mild drought stress was sufficient to reset the epigenomes and result in phenotypically similar progeny. The authors conclude that mild drought has no transgenerational effects in *A. thaliana*. They state that there is a “growing body of evidence against transgenerational epigenetic changes being a predictable and common response of plants to changes in the environment”. They also hedge their conclusions by noting that a documentable transgenerational effect may result from a more dramatic stress or perhaps from a combination of stresses.

The value of making the distinction between inter- and transgenerational effects when considering epigenetic inheritance, especially where differential maternal provisioning is clearly the determining factor, is illustrated by studies of these effects in durum wheat (*Triticum durum* Desf.) in response to a combination of drought and heat stress and in the soybean in response to drought stress (Wijewardana et al., 2019; Liu et al., 2021). In both studies, only the F_1_ of stressed parents was evaluated, and performance measures were only for seed and seedling performance. In both studies negative intergenerational effects were observed; seeds obtained from stressed parents had reduced germination rates and reduced seedling vigor. In both studies, the casual factor clearly reduced maternal provisioning resulting in reduced seed size, viability and performance yet the effects were referred to as transgenerational. However, both inter- and transgenerational effects might have been observed if performance at the adult stage of the F_1_, or in the F_2_, were evaluated.

Transgenerational adaptation may be conditioned by a unique type of siRNAs, the 21- and 24-nt “phased siRNAs” (phasiRNAs) that accumulate during male gametogenesis in pollen (Yadava et al., 2021). Pollen accumulation of phasiRNAs was reported in maize (Zhai et al., 2015) and in rice (Fan et al., 2016) and is now known to be a common phenomenon in angiosperms (Xia et al., 2019). Their potential agronomic importance is illustrated by the fact that hybrid rice production uses the *photoperiod-sensitive genic male sterility 1* (*PMS1*) locus which encodes a long-noncoding RNA that is processed into 21-nt phasiRNAs that accumulate in pollen (Fan et al., 2016). Yadava et al. (2021) studied the environmentally-sensitive male sterility *outer cell layer 4* (*ocl4*) locus in maize. They found that in one derivative line male fertility restoration by warm temperatures was enhanced as compared with other lines of similar genotype, that this was mediated by the elevated production (as compared with sterile counterparts) and pollen accumulation of a 21-nt phasiRNA, and that this conditioned fertility was perpetuated over several generations but apparently only when pollen maturation occurred under warm temperatures. Thus, it is debatable whether or not this is a case of epigenetically-mediated transgenerational inheritance, according to the strict rules discussed above. Looking forward, one attractive hypothesis concerning pollen-accumulated phasiRNAs is that they could possibly play a role in transmitting epigenetic code to the next generation following pollination.

While studies of the role of epigenetic inheritance in adaptation is by definition and interest largely focused on adaptation to a given stress or environmental change, in crop production it is of equal practical importance that the epigenetic changes may enhance progeny crop performance in production environments that are optimal or relatively non-stressful. Perhaps this is what has been demonstrated *via* the *MSH1-*RNAi approach of the Mackenzie group (Kundariya et al., 2020; Yang et al., 2020; Ketumile et al., 2022). In that approach, a form of metabolic stress is imposed *via* silencing of *MSH1* using RNAi. This induces methylome repatterning, some of which is heritable for several generations in the absence of *MSH1* silencing, and some of which conditions enhanced vigor in relatively non-stressful environments (Kundariya et al., 2020).

As compared with the *MSH1*-RNAi approach, a more low-tech and thus more widely available approach world-wide would be to simply expose parent plants in seed production programs to a well-designed and inexpensive-to-impose environmental change or stress treatment such as differing levels of drought or varying macronutrient supply. The classic example of this is the demonstration that in flax (*Linum usitatissimum* L.), seed obtained from parents grown with varying levels of the macronutrients nitrogen, phosphorus, and potassium, produced plants which provided substantially more yield when grown with fertilizer than did seed obtained from plants not grown with fertilizer (Durrant, 1958). Although quite variable, this effect was reproducible and clearly not entirely attributable to maternal effects. Interestingly, efforts to understand the molecular basis of this apparently epigenetic phenomenon invoked the genetic mechanism of DNA rearrangement due to TE mobilization (Cullis, 1990). Is this an example of “paradigm-lock”: an attempt to explain an epigenetic phenomenon in terms of the more widely accepted genetic paradigm?

Another alternative to the *MSH1-*RNAi approach of the Mackenzie group that has great potential and that avoids the need for plant transformation would be to use a topically-applied “stress mimic” spray treatment for parents in seed production programs. For example, one might spray parents in seed production fields with a double-stranded RNA (dsRNA)-containing solution that results in RNAi suppression of selected target genes, such as *MSH1*, that results in transgenerational enhancement of crop performance. Over the last decade this type of approach has been developed and documented to work intragenerationally for the management of plant viral and fungal pathogens (Tenllado and Dıaz-Ruız, 2001; Dubrovina et al., 2019; Werner et al., 2020; Gebremichael et al., 2021). This type of treatment can have epigenetic effects. Dubrovina et al. (2019) demonstrated that topical application of dsRNA targeting *Enhanced Green Fluorescent Protein* (*EGFP*) and *Neomycin phosphotransferase II* (*NPTII)* reporters in *A. thaliana* resulted in DNA methylation of target gene sequences and regions. Key advantages of this approach over the use of stress treatments is its ease of use and that it would avoid negative impacts of stress treatments on the parent plants, insuring optimal seed yield and seed quality. What if simply spraying plants in a seed production field, or male parents in a hybrid seed production field, with a solution containing various dsRNAs, can result in a transgenic enhancement of productivity by 10% to 25%?

Simple use of parental treatment or stress in seed production to produce crops with enhanced performance in optimal or non-stressful environments would represent an “appropriate technology” (Schumacher, 1973). It would be low-tech, low-cost, widely accessible and available in both developed and developing economies. Furthermore, it would not require the use of genetic modification and thus would avoid the regulatory hurdles discussed above.

A distinction informative to this discussion is between the role of epigenetic phenomena in crop breeding versus in crop production. Perhaps what matters is not the mechanism of inheritance but rather its duration. Crop breeding may depend more heavily on longer-term inheritance whereas crop production may simply take advantage of shorter-term phenomena, such as the above cases of transgenerational inheritance. Isn’t taking advantage of parental treatments to enhance or stabilize yields or field performance a production strategy rather than a breeding strategy? Utilizing hybrid seed in crop systems is also a production strategy, albeit one that then requires breeding for optimization. Thus, the recent studies that have revealed a role for epigenetics in the phenomenon of heterosis (Groszmann et al., 2013; Dapp et al., 2015; Kawanabe et al., 2016; Fujimoto et al., 2018; Lauss et al., 2018; Miyaji and Fujimoto, 2018), have in fact revealed a role for epigenetics in an important production strategy. A third example of this distinction is the case of *Karma* and the mantling trait in the oil palm (Ong-Abdullah et al., 2015). In that case, understanding the epigenetic basis of the trait allows for the more efficient selection of lines for production rather than for breeding. In light of this distinction, the list of cases clearly describing the novel use of epigenetics specifically in crop breeding might be fairly limited. A recent review of maternal effects and intergenerational adaptation in response to biotic and abiotic stress concludes that this phenomenon should be considered in the establishment of forestry plantations (Vivas et al., 2020). This is clearly a proposal to use epigenetics to enhance production, rather than in breeding.

Since exposure to stress or environmental change induces heritable epigenome-mediated changes in the transcriptome, some of which may impart enhanced stress-tolerance or enhanced vigor, might not these induced changes in gene expression be the subject of paramutation? This is supported by the observation that a role for transgenerational memory in paramutation has been previously documented (Mikula, 1995; Bente et al., 2021). Furthermore, since the phenomenon of paramutation appears to occur at numerous loci throughout the genome (Li et al., 2013; Regulski et al., 2013; Gouil and Baulcombe, 2018), this form of non-Mendelian inheritance might function to rapidly fix (make homozygous) these stress-induced epigenome changes in all progeny of the stress-exposed parents (**Figure 5** bottom). If this “pseudo-Lamarckian”, paramutation-mediated process of fixing adaptive stress-induced epigenomic changes occurs, this would have substantial significance in crop breeding programs and would have significant implications for how such changes are followed using molecular assays that purport to map and follow such changes following out-crossing.

## Future perspectives

The stable inheritance of traits is important for the use of epialleles in breeding perhaps more so than for applications in crop production *per se*. Unlike DNA sequence changes, DNA methylation will lead to complicated DNA methylation states in crossbreeding populations, but it still has potential to make new and desirable phenotypes that cannot be produced by genetic diversity. Marker assisted selection (MAS) and genomic selection, which exploit genetic variation, have made a significant contribution to the efficiency of breeding. In order to apply the same methods to epimutation, it is necessary to develop a method for selecting epialleles that is high-throughput. Inducing epimutation by chemical treatment or epigenomic editing may be able to produce new traits. Since DNA methylation is associated with gene expression, change of DNA methylation of regions that regulate gene expression such as *cis*-elements may induce new gene expression associated with new phenotype.

It is clear that taking practical advantage of the potential of epigenetics in crop breeding is in its infancy. While a great deal of interest and enthusiasm has been generated, as evidenced by the numerous review articles addressing the potential of this new science and technology (see for example: Springer and Schmitz, 2017; Kawakatsu and Ecker, 2019; Dalakouras and Vlachostergios, 2021; Turcotte et al., 2022), there are relatively few cases of successful application of this new science to plant breeding, with “successful application” being defined as commercial release of a new cultivar or hybrid. This could be due to the inherent instability and variability of epigenetic programming and the difficulty in identifying precise treatments and approaches for its utilization. It could also reflect the lengthy period of time required to go from the discovery or development of a valuable trait to the commercial release of a new cultivar or hybrid developed utilizing that trait, a period estimated to range from five to 20 years (Ahmar et al., 2020). Furthermore, the novelty of the science and technology of epigenetics would further delay occurrence and documentation of such successful outcomes. With that said, the application of new knowledge concerning epigenetic inheritance may ultimately result in a substantial enhancement in crop productivity world-wide.

However, it is possible that full appreciation of the potential of epigenetics and its subsequent application to crop breeding and production has been somewhat limited by the tendency to attempt to fit epigenetic inheritance into classical models of Mendelian inheritance and evolution, such as those underlying the “modern synthesis” that marries Mendelian inheritance with molecular biology (Laland et al., 2014; Pembrey, 2015). For example, the numerous reviews of the potential of epigenetics in crop improvement cited above typically focus on methods for mapping and breeding based on Mendelian modes of inheritance, whereas non-Mendelian modes of induction and inheritance of epigenomic variation are common for epigenome-determined traits. These reviews typically do not highlight or stress how non-Mendelian modes of induction and inheritance of epigenome-determined traits can be taken advantage to rapidly enhance crop yields, such as the potential use of transgenerational transmission of stress-induced change, or the potential of paramutation to rapidly fix adaptive changes in F_2_ progeny.

Pecinka and Scheid (2012) proposed a set of criteria that should be met to establish epigenetic yet chromatin-mediated transfer of stress adaptation to offspring in plants. Application of such criteria is of great benefit to the field, assuring that claims of longer-term inheritance of epigenetic variants are valid. However, focusing on fitting epigenetic phenomena into modern synthesis models may also limit appreciation of the potential for what may only be very short-term phenomena limited to one or two generations, or phenomena that involve siRNA transmission through pollen, in crop breeding and production. To illustrate this, we can consider the discussion of the role of epigenetics in human evolution by Pembrey (2015). Pembrey (2015) points out that if a doctor has a patient in his/her office that has an illness, such as diabetes, that may have been mediated by transmission of an epigenetic state from his/her parents or grandparents, then dealing with the patient’s illness in the present is more important than fully understanding the role of epigenetics across many generations in evolution. This is an exact parallel to the question of intra- and transgenerational adaptation to stress in crop plants. Does it really matter whether or not epigenetic inheritance plays a central role in evolution, or is unstable across many generations, if in fact one can substantially enhance and stabilize yields by appropriately stressing parents?

## Author contributions

KT, RF, VR, and KO prepared the manuscript text and figures. ESD and KO revised and edited the manuscript. ESD made critical comments to improve the manuscript contents and also edited the English. All authors contributed to the article and approved the submitted version.

## Funding

This work was funded by Grant-in-Aid for Early-Career Scientists (20K15504) of Japan Society for the Promotion of Science (JSPS) granted to KT, and Grant-in-Aid for Challenging Research (Exploratory) (20K21313) granted to RF and KO, and Kobe University Strategic International Collaborative Research Grant (Type B Fostering Joint Research), Fund for the Promotion of Joint Research (16KK0171), Grant-in-Aid for Scientific Research (B) (19H02947, 22H02338) of Japan Society for the Promotion of Science (JSPS) granted to RF.

## Conflict of interest

The authors declare that the research was conducted in the absence of any commercial or financial relationships that could be construed as a potential conflict of interest.

## Publisher’s note

All claims expressed in this article are solely those of the authors and do not necessarily represent those of their affiliated organizations, or those of the publisher, the editors and the reviewers. Any product that may be evaluated in this article, or claim that may be made by its manufacturer, is not guaranteed or endorsed by the publisher.
