# Corrigendum: Will epigenetics be a key player in crop breeding?

**DOI:** 10.3389/fpls.2023.1157933

**Published:** 2023-03-03

**Authors:** Kaoru Tonosaki, Ryo Fujimoto, Elizabeth S. Dennis, Victor Raboy, Kenji Osabe

**Affiliations:** ^1^ Kihara Institute for Biological Research, Yokohama City University, Yokohama, Japan; ^2^ Graduate School of Agricultural Science, Kobe University, Kobe, Japan; ^3^ Commonwealth Scientific and Industrial Research Organisation (CSIRO) Agriculture and Food, Canberra, ACT, Australia; ^4^ School of Life Sciences, Faculty of Science, University of Technology Sydney, Ultimo, NSW, Australia; ^5^ Independent Researcher Portland, Portland, OR, United States; ^6^ Institute of Scientific and Industrial Research (SANKEN), Osaka University, Osaka, Japan

**Keywords:** DNA methylation, breeding, intergenerational inheritance, transgenerational inheritance, epigenetics, epiallele, epigenome editing, paramutation

**Error in Figure/Table**

In the published article, there was an error in **Figure 5** as published. The ‘*’ on the ‘*SR’* was incorrectly positioned. The corrected **Figure 5** and its caption appear below.

**Figure 5:**
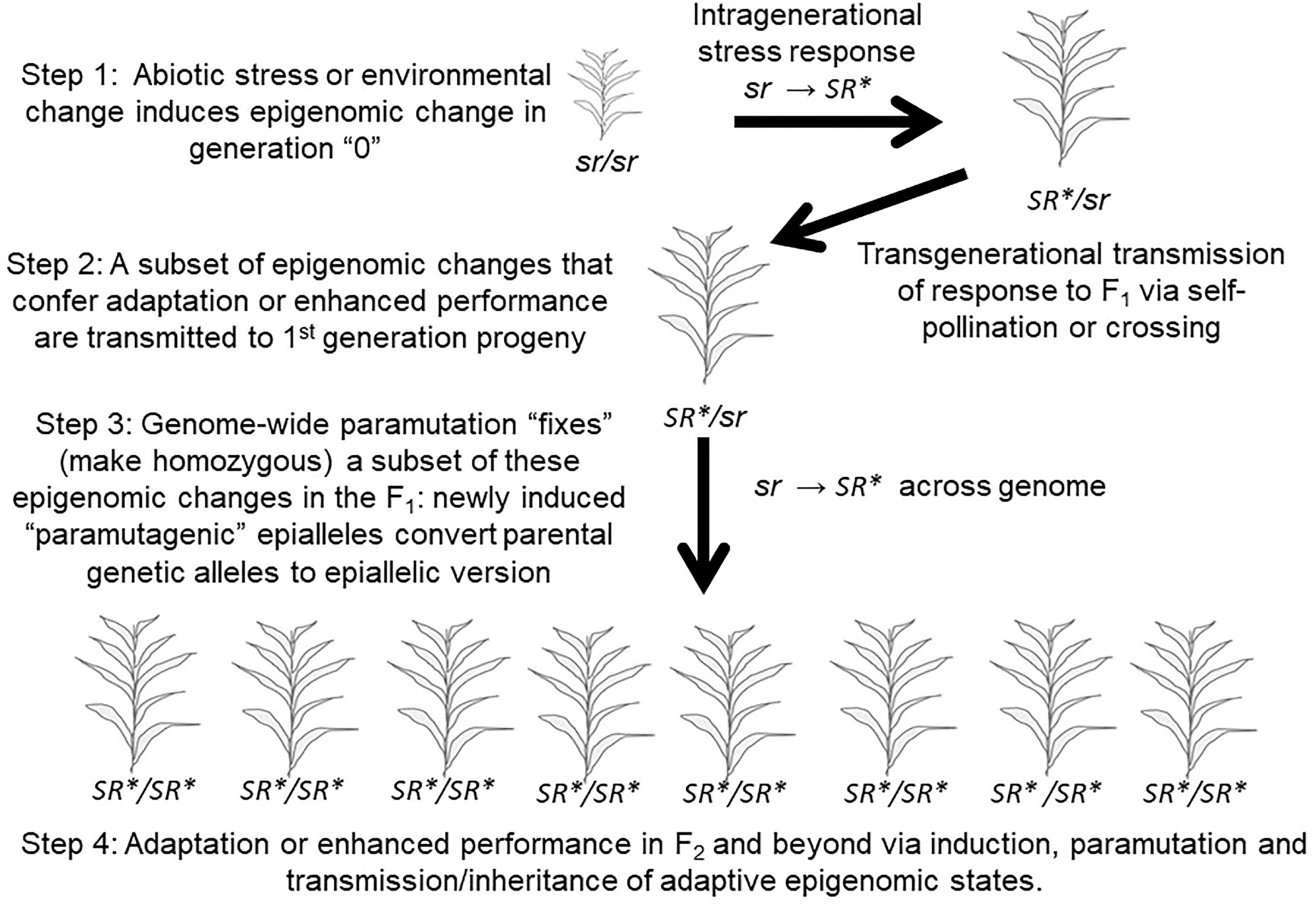


The authors apologize for this error and state that this does not change the scientific conclusions of the article in any way. The original article has been updated.

